# Serious physical assault and subsequent risk for rehospitalization in individuals with severe mental illness: a nationwide, register-based retrospective cohort study

**DOI:** 10.1186/s12991-021-00358-y

**Published:** 2021-09-18

**Authors:** Karolina Mlada, Tomas Formanek, Jan Vevera, Klara Latalova, Petr Winkler, Jan Volavka

**Affiliations:** 1 Department of Public Mental Health, National Institute of Mental Health, Klecany, Czech Republic; 2 Department of Psychiatry, Faculty of Medicine, University Hospital in Pilsen, Charles University, Prague, Czech Republic; 3 EpiCentre, Department of Psychiatry, University of Cambridge, Cambridge, UK; 4 Department of Psychiatry, First Faculty of Medicine, Charles University in Prague and General University Hospital in Prague, Prague, Czech Republic; 5 Institute for Postgraduate Medical Education Prague, Prague, Czech Republic; 6 Department of Psychiatry, University Hospital Olomouc, Olomouc, Czech Republic; 7 Faculty of Medicine and Dentistry, Palacky University Olomouc, Olomouc, Czech Republic; 8 Health Service and Population Research Department, Institute of Psychiatry, Psychology and Neuroscience, King’s College London, London, UK; 9 Department of Psychiatry, New York University School of Medicine, New York, Emeritus USA

**Keywords:** Victimization, Assault, Severe mental illness, Hospitalization, Violence, Aggression

## Abstract

**Background:**

Victimization is associated with worse social and clinical outcomes of individuals with severe mental illness (SMI). A relapse of SMI may be one of the clinical consequences of assaultive trauma. As far as we know, there is no published study that analyzes nationwide health registers to assess the risk of SMI rehospitalization following assault.

**Aim:**

We aimed to assess whether exposure to assault is associated with an increased risk of psychiatric hospitalization in those with SMI.

**Methods:**

We utilized data from the Czech nationwide registers of all-cause hospitalizations and all-cause deaths. We defined exposed individuals as those discharged from a hospitalization for SMI between 2002 and 2007, and hospitalized for serious injuries sustained in an assault in the subsequent 7 years. For each assaulted individual, we randomly selected five counterparts, matched on SMI diagnosis, age and sex, who were not assaulted in the examined time period. We used mixed effect logistic regression to assess the effect of assault on the risk of SMI rehospitalization within the following 6 months. We fitted unadjusted models and models adjusted for the number of previous SMI hospitalizations and drug use disorders.

**Results:**

The sample consisted of 248 exposed and 1 240 unexposed individuals. In the unadjusted model, assaulted individuals were almost four times more likely to be rehospitalized than their non-assaulted counterparts (odds ratio (OR) = 3.96; 95% CI 2.75; 5.71). After adjusting for all covariates, the OR remained threefold higher (OR = 3.07; 95% CI 2.10; 4.49).

**Conclusion:**

People with a history of SMI hospitalization were approximately three times more likely to be rehospitalized for SMI within 6 months after an assault than their non-assaulted SMI counterparts. Soon after a person with SMI is physically assaulted, there should be a psychiatric evaluation and a close follow-up.

**Supplementary Information:**

The online version contains supplementary material available at 10.1186/s12991-021-00358-y.

## Introduction

### Severe mental illness and relapse

Severe mental illness (SMI) is defined as schizophrenia, schizoaffective disorder, bipolar disorder, or major depressive disorder. It leads to a substantive reduction of quality of life, affecting both individuals and their caregivers [1, 2], and is associated with a large societal cost. [3, 4] Recurrent SMI hospitalizations drive up the cost of psychiatric care, and can further impair quality of life [5]. Relapses in individuals with SMI are associated with an increased risk of long-term disability and suicide attempts [6].

### SMI and risk of victimization

As confirmed by an influential meta-analysis, individuals with SMI experience victimization disproportionally more often than the general population [7]. Victimization may take various non-violent or violent forms [1]. Specific risk factors for victimization in adults diagnosed with psychotic disorders include drug or alcohol abuse, a high overall psychotic symptom score, homelessness, perpetration of a crime, and negative life experiences, such as previous adult victimization or child maltreatment [7, 8].

### Victimization and risk of impaired course of SMI

We have seen that SMI can affect the risk of victimization. However, the relationship between SMI and victimization may also work in the opposite direction: victimization may affect the time course of SMI. Preliminary evidence from several sources based largely on convenience samples of patients suggests that victimization may make the SMI worse. The likelihood of remission was found to be decreased in people with bipolar disorder who suffered assaultive trauma [9]. In men with schizophrenia, victimization was associated with a general impairment of functioning [10], while victimization of people with mental disorders was followed by an increased incidence of depression, anxiety and panic attacks when compared to the control group [11]. Recent violent victimization can also independently increase the risk of violence in individuals with schizophrenia [12]. In addition, women with schizophrenia victimized by sexual assault demonstrated an elevated risk for annual rehospitalization following the assault when the assault was preceded or accompanied by drug use [13]. However, to our knowledge, there is no published assessment of risk for psychiatric rehospitalization after victimization in individuals with SMI based on epidemiological data from nationwide health registers. Such data could be useful for prevention or reduction of relapse risk in future victimized patients with SMI.

### The hypothesis

To formally test a relationship between physical victimization and relapse, we hypothesized that during 6 months after an assault, the risk for a psychiatric hospitalization of people with SMI living in the community would be greater than that of their non-assaulted counterparts. The aim of the present study was to examine this hypothesis using data from Czech nationwide health registers.

## Methods

### Data

We used data from two nationwide health registers, maintained by the Czech Institute of Health Information and Statistics: 1. the register of all-cause hospitalizations; and 2. the register of all-cause deaths. Both registers are described in-depth elsewhere [14]. Briefly, data from the register of all-cause hospitalizations are available from 1994, with approximately 2.3 million hospital records per year, of which around 2.5% are related to mental disorders. It contains diagnoses coded as per the International Statistical Classification of Diseases and Related Health Problems (ICD-10), information regarding admission and discharge and basic socio-demographic information. The coding procedure is briefly described in the Additional file 3. The register of all-cause deaths also goes back to 1994 and consists of basic socio-demographic information and primary cause of death according to the ICD-10. In the present study, we used data covering the time period from January 1st, 1995 to December 31st, 2017. The approval for this study was obtained from the ethics committee of the National Institute of Mental Health, Czech Republic (number 105/18). Individual informed consent was not obtainable, since this is an observational study using anonymized data from nationwide health registers.

### Sample

**Fig. 1:**
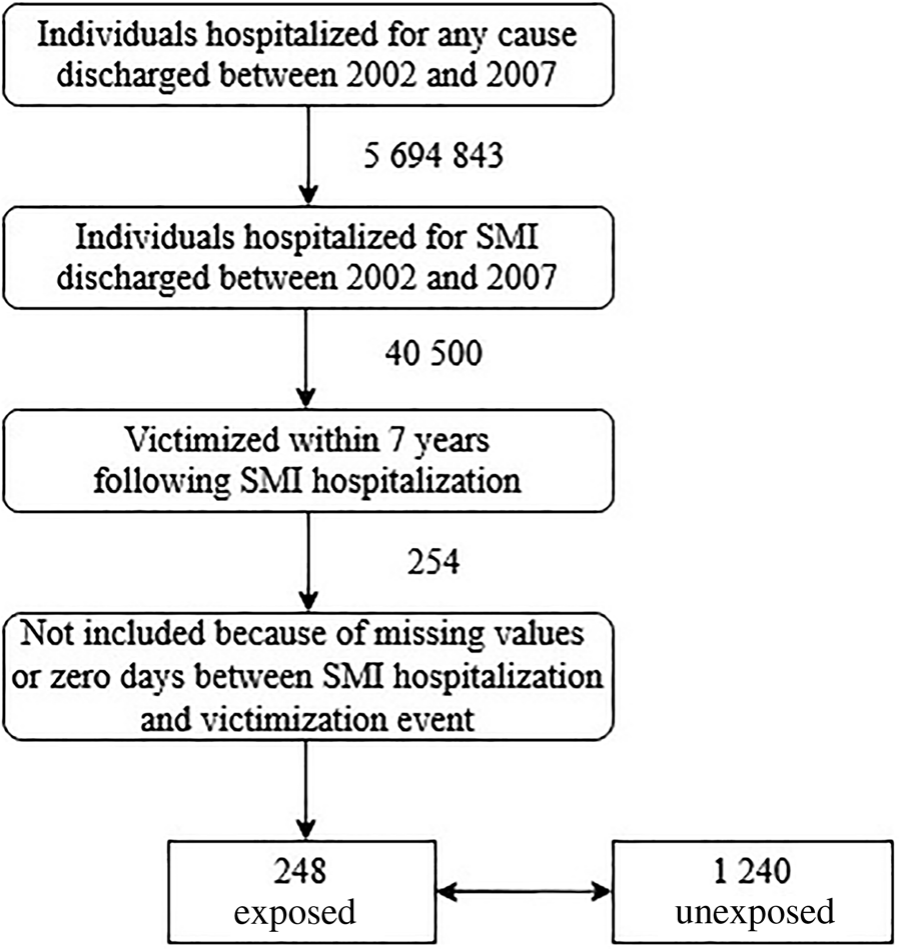
Flowchart of sample creation

The construction of the sample is illustrated in Fig. 1. We identified individuals hospitalized for SMI (ICD codes F20, F25, F31, F32, F33; details in Additional file 2) and discharged between 2002 and 2007 from the register of all-cause hospitalizations (*n* = 40,500). Identical or similar definitions of SMI have been used by other investigators [15–17]. Then, we assessed whether individuals were admitted due to injury sustained in an assault in 7 years after SMI hospitalization (ICD codes X93–95, X99, Y00–Y05). Using this procedure, we obtained 254 individuals with a history of hospitalization for SMI who were assaulted. One of these individuals had no valid information on age and five individuals were discharged from SMI hospitalization the same day the assault occurred, thus, we excluded them from further analysis. The final sample consisted of 248 individuals, and throughout the text we refer to them as exposed individuals.

**Fig. 2:**
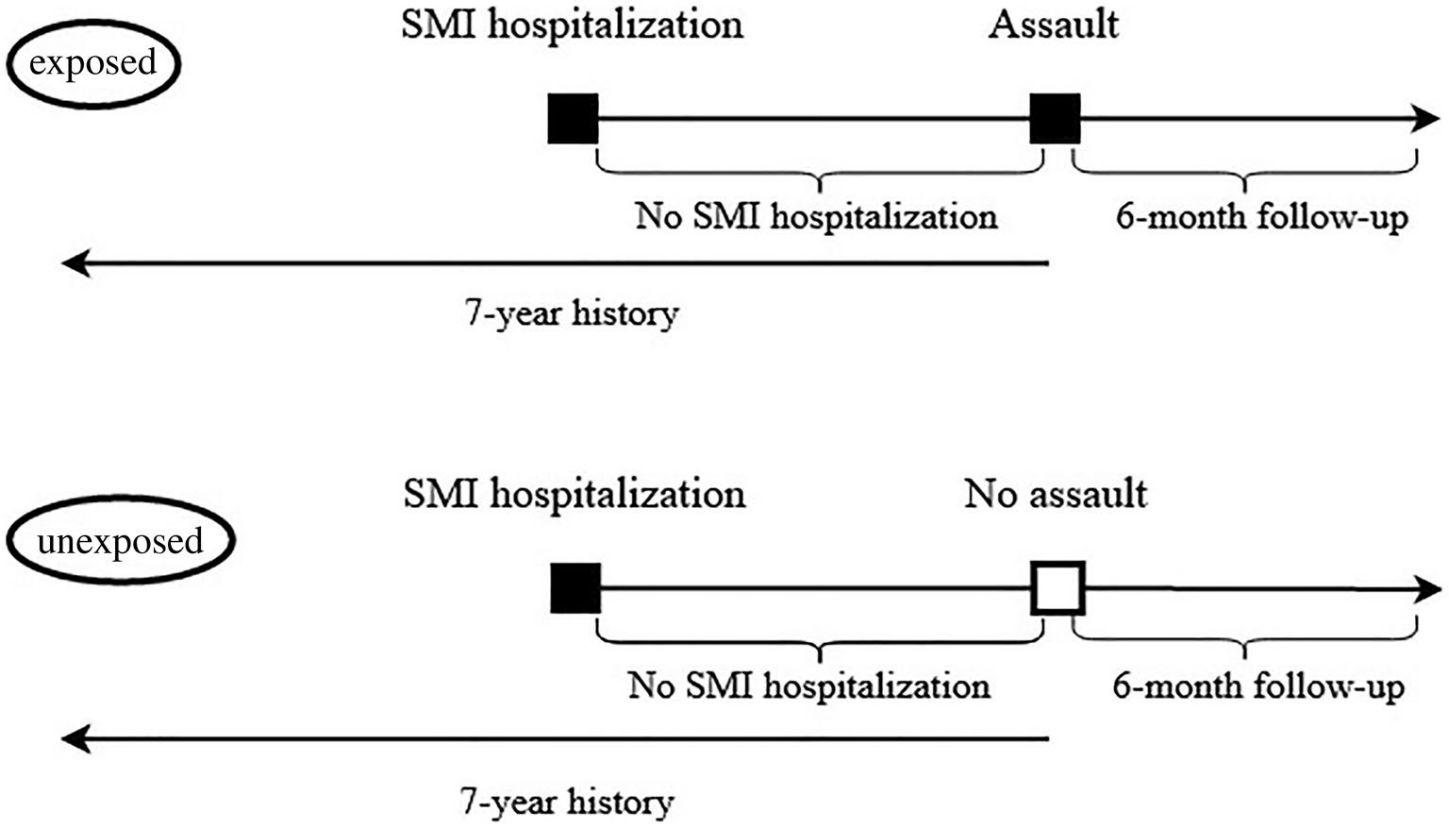
Matching procedure

To minimize the differences between those exposed and those not exposed to assault, we performed a matching on age (with the possibility of non-exact match, up to a difference of +—3 years), gender, and the SMI diagnosis on the exposed individual’s last SMI hospitalization before exposure to assault. We matched these characteristics in particular as they are understood to be the key sociodemographic and clinical characteristics. In addition, for each exposed individual, we calculated the time difference (in days) between the discharge date from the last SMI hospitalization and the admission date of the assault-related hospitalization, and excluded the matching counterparts who experienced hospitalization for SMI in the equivalent of this time period (Fig. 2). Then, using data from the register of all-cause deaths, we included only those individuals who did not die before the end of study period. We randomly selected 5 unexposed individuals for each exposed one, ending up with 1 240 unexposed individuals. We used a 5:1 ratio given the large number of potential matching candidates, while also reflecting that the use of a higher ratio would likely translate to only a marginal gain in efficiency [18].

### Outcomes

#### Rehospitalization

We used rehospitalization as the main outcome as it is widely used as a proxy to define relapse in people with SMI [6]. In assaulted individuals, SMI rehospitalizations were assessed in a time window of 6 months after the assault-related hospitalization. As per the definition of the cohorts, the unexposed individuals did not experience an assault-related hospitalization; thus, for them, we established a 6-month follow-up window which emulated the one used for the exposed individuals. First, for each exposed individual, we calculated the time difference between the discharge date from the last SMI hospitalization preceding the assault and the discharge date of the assault-related hospital stay. Second, we added this time difference (expressed in days) to the unexposed counterpart’s discharge date on the matching SMI hospitalization. Finally, we assessed the presence of any SMI rehospitalization in 6 months following the obtained timepoint.

### Covariates

#### Hospitalization for drug use disorder in 7 years prior to the start of the follow-up

Drug use is associated with increased risk for assault in people with mental disorders [7], thus, we considered the history of drug use disorders (DUD) as a potential confounder. In assaulted individuals, we assessed hospitalizations for DUD (ICD codes F11, F13, F14, F15 and F16) in 7 years before the assault-related hospitalization. In unexposed individuals, we computed the matching assaulted individual’s number of days from the end date of the last SMI hospitalization to the admission date of the assault-related hospitalization. We added this time difference to the unexposed individual’s discharge date, and assessed the presence of DUD in 7 years preceding this timepoint. We created a binary coded variable, with no DUD hospitalization being the reference category.

#### Number of SMI hospitalizations in 7 years prior to the start of the follow-up

To control for potentially differing histories of SMI hospitalizations between assaulted and non-assaulted cohorts, we computed a variable denoting the individuals’ history of SMI hospitalizations. We established the number of SMI hospitalizations (any of ICD codes F20, F25, F31, F32 and F33) for assaulted individuals as the sum of SMI hospitalizations in the 7-year period prior to the date of admission to assault-related hospitalization. For non-assaulted individuals, we established the history of SMI hospitalizations using the same procedure as described above for the history of DUD hospitalization.

### Statistical methods

We computed descriptive statistics of the sample, expressed as counts (n) with proportions (%) for categorical variables, means (M) with standard deviations (SD) for normally distributed variables, and medians with interquartile range (IQR) for non-normally distributed variables. We used Chi-square test (for comorbid DUD) and the Mann–Whitney *U* test (for the number of previous SMI hospitalizations) to assess the differences between cohorts. To assess the association between assault-related hospitalization and subsequent psychiatric hospitalization, we employed mixed effects logistic regression, with exposed individuals being set as random intercepts. We computed three models, overall: (1) the crude model containing only a variable referring to assault, (2) a model adjusted for the number of SMI hospitalizations in 7 years prior to the start of the follow-up, and 3. a model adjusted for the number of SMI hospitalizations and comorbid DUD in 7 years prior to the start of the follow-up. We considered associations with *p* < 0.05 as statistically significant. We performed the data analysis using Microsoft Access 2013 and R statistical programming language (version 3.6.0). We followed the STROBE guidelines (see Additional file 1).

## Results

### Description of the sample

The detailed description of the sample is provided in Table 1. The final dataset consisted of 248 assaulted and 1240 non-assaulted individuals (mean age 36 years, 63% males for both cohorts). The vast majority of exposed individuals (201; 81%) experienced an assault by bodily force (ICD code Y04) and 50% of exposed (*n* = 125) suffered intracranial injury (ICD code S06). The largest proportion of individuals had schizophrenia as their diagnosis on their last SMI hospitalization (37%), followed by major depressive disorder (28%), schizoaffective disorder (16%), recurrent depressive disorder (12%) and bipolar disorder (8%). Approximately 6% of assaulted individuals were hospitalized for DUD in the past, whereas in non-assaulted it was 2%. This difference was statistically significant (*p* value < 0.01). In addition, we observed a statistically significant higher number of previous SMI hospitalizations in assaulted individuals (median = 2, IQR = 2 and median = 1, IQR = 1, *p* value < 0.001), when compared to non-assaulted ones.

**Table 1:** Characteristics of the sample

	Exposed	Unexposed
Age on last SMI hospitalization, mean (SD) ^a^	36.34 (12.33)	36.34 (12.31)
Males, % (*n*) ^a^	62.90 (156)	62.90 (780)
Last SMI diagnosis before the start of follow-up, % (*n*) ^a^		
Schizophrenia F20.0–F20.9	36.69 (91)	36.69 (455)
Schizoaffective disorder F25.0–F25.9	15.73 (39)	15.73 (195)
Bipolar disorder F31.0–F31.9	7.66 (19)	7.66 (95)
Major depressive disorder, single episode F32.0–F32.9	27.82 (69)	27.82 (345)
Major depressive disorder, recurrent episode F33.0–F33.9	12.10 (30)	12.10 (150)
Comorbid DUD in the last 7 years before victimization, % (*n*)^b^ **	5.65 (14)	1.94 (24)
Number of SMI hospitalizations in the last 7 years before victimization, median (IQR)^b^ **	2 (2)	1 (1)

^a^The unexposed individuals were matched with exposed on gender, age and last SMI diagnosis; therefore, the distribution on these variables is identical

For the comparison on comorbid DUD, a Chi-square test was used. For the comparison on number of SMI rehospitalizations, a Mann–Whitney *U* test was used

^b^The unexposed individuals did not experience an actual victimization-related hospitalization. Thus, the presence of DUD and the number of SMI hospitalizations in unexposed individuals was assessed using a generated time windows copying the time windows of the exposed individuals with which they were matched

^**^p value of the test was lower than 0.01

### Mixed effects logistic regression

Detailed results are provided in Table 2. The results of the crude model indicate that experiencing assault was associated with increased odds for subsequent SMI rehospitalization (OR 3.96; 95% CI 2.75; 5.71). When adjusting for the number of previous SMI hospitalizations, the effect size was slightly mitigated, nevertheless the trend remained unchanged (OR = 3.10; 95% CI 2.13; 4.53). Similar results were obtained after the inclusion of previous DUD-related hospitalizations, with the effect size of assault on subsequent psychiatric hospitalization being further attenuated (OR = 3.07; 95% CI 2.10; 4.49).

**Table 2:** Mixed effects logistic regression with odds ratios of being rehospitalized in the 6 months following assault

	Model 1 OR (95% CI)	Model 2 OR (95% CI)	Model 3 OR (95% CI)
Experienced assault	3.96 (2.75; 5.71) ***	3.10 (2.13; 4.53) ***	3.07 (2.10; 4.49) ***
Number of SMI hospitalizations in the last 7 years before assault^a^	–	1.27 (1.20; 1.35) ***	1.27 (1.20; 1.35) ***
Comorbid DUD in the last 7 years before assault^a^	–	–	1.31 (0.52; 3.33)

^a^The unexposed individuals did not experience an actual assault-related hospitalization. Thus, the presence of DUD and the number of SMI hospitalizations in unexposed individuals was assessed using a generated time windows copying the time windows of the exposed individuals to which they were matched

*** *p* value of the test was lower than 0.001

## Discussion

### What is new

In this register-based retrospective cohort study, we found that individuals with SMI who experienced an assault-related hospitalization had an approximately three times higher risk to be hospitalized for SMI within the subsequent 6 months than their non-assaulted counterparts. In general, the risk of SMI relapse and rehospitalization is increased by comorbid DUD, [7, 13, 19] and comorbid DUD was detected significantly more frequently in assaulted individuals than in non-assaulted ones. Nevertheless, our analysis has demonstrated that our principal finding was not due to potential confounding effects of DUD. Likewise, the number of previous SMI hospitalizations was greater in assaulted individuals, and the inclusion of this variable in the logistic regression model further reduced the effect of assault.

### Comparison with existing literature

Our observation of a relative increase of hospitalization risk after assault is comparable with a number of studies, all of which used designs that differed from ours. A principal difference between our study and other published investigations on this topic is the sample selection. We have included only assaults that were severe enough to require hospitalization. Comparable studies used less stringent definitions of violent victimization [20, 21].

Rabinovitz et al. reported a statistically significant contribution of comorbid DUD to the elevation of the rehospitalization risk after assault of people with schizophrenia [13]. We did not find that in our study, however, these differences might be at least partially due to methodological differences, since Rabinovitz et al. had access to information contemporaneous with the assault, while our information was historical and register-based. Our findings are consistent with those published by Neria et al. [9], who assessed trauma histories in a cohort of people with first episode of bipolar disorder, and found that trauma affected the course of illness: exposed individuals were more symptomatic than unexposed, and were less likely to remit than the unexposed. The principal contribution of our new findings to the existing literature consists in the quantitative estimate of the risk of SMI relapse following victimization of SMI patients.

### Theoretical aspects and interpretation

The causal mechanisms of the difference in hospitalization risks in assaulted and non-assaulted individuals are not clear yet. Some assaults might have been provoked by an individual’s behavior influenced by psychotic symptoms heralding an incipient relapse and hospitalization. Unintentional major trauma (as opposed to assault, which is intentional by definition) was associated with a significant increase of hospital admission for new or pre-existing mental health diagnoses in a population-based cohort study [22]. Thus, it is possible that factors other than SMI, for example the stress of physical trauma, were at least partly responsible for the difference in the hospitalization risks we observed. Mueser hypothesized that traumatic experiences of people with SMI might elicit posttraumatic stress disorder (PTSD) which in turn worsen the course of SMI by direct and indirect effects [23]. A direct effect would be PTSD symptoms acting as stressors on vulnerable people with SMI, leading to more SMI symptoms and relapse. An indirect effect would be associated with the use of alcohol or drugs to cope with PTSD symptoms, resulting in relapse and rehospitalization. PTSD-related personal problems would be another example of indirect effect of PTSD on functioning.

### Practical implications

Our results have both clinical and public health implications. To mitigate or prevent the effects of a physical assault on the mental health of individuals with SMI, and in particular to prevent a relapse, the victim should be evaluated by a mental health professional. As victimized people with SMI are at elevated risk for relapse, the mental health professional should provide support and schedule a follow-up. Antipsychotic medication may need adjustment, and adherence should be stressed. The circumstances of the assault should be explored, and strategies for avoiding repeated assault should be discussed. The evaluation should include inquiry about the person’s own violent behavior, since victimization and perpetration of violence increase each other’s risk [8, 20, 24]. If the person with SMI is also a perpetrator of violence, appropriate treatments using cognitive behavioral approach and conflict resolution strategies should be added [25]. In all cases of victimization, the psychiatrist should inquire about substance use and address it in the treatment plan if necessary. From a public health perspective, studies looking at cost-effectiveness of enhanced psychiatric care for victimized people with SMI are encouraged. Compared to in-patient care, care in the community has been found to be notably more cost-effective in the Czech Republic as well as in other countries [26, 27]. Interventions that could reduce rehospitalizations would help to use scarce resources more effectively. At the very least, a history of victimization has important risk implications regarding the prognosis of individuals with SMI and efforts are needed to ensure that this information is collected routinely when assessing persons with major mental disorders.

### Strengths of the study

This study benefited from the use of data from nationwide registers, consisting of essentially all hospitalizations in a period of more than 20 years, effectively eliminating the selection biases inherent in prospective cohort studies. Second, we randomly matched the assaulted individuals with unexposed individuals on several socio-demographic and clinical characteristics, assuring they had approximately the same profile. This procedure increased the likelihood that the differences between assaulted and non-assaulted individuals were not because of the matching procedure itself, but because of real differences between the cohorts. Third, the definition of assault was clear and objective by requiring injury severe enough to lead to hospitalization. There were no false positives. Finally, our study has external validation as evidenced by the observation of the victimization effect on the time course of bipolar disorder [9], and in a report on mental health outcome of major traumatic injury [22].

### Limitations of the study and scope for future studies

Although this study had several strengths, its limitations also need to be addressed. First, the definition of assault used in this study did not include victimization incidents that were less serious and did not require hospitalization. Because of this, the results may not be generalizable to less severe incidents. To an extent, our report shares this limitation with a recent major study that also focused only on assault resulting in injuries requiring medical care [21]. Second, the health registers contain information on psychiatric diagnoses, but no information on individuals’ psychiatric symptoms, duration of the illness, treatment, or treatment adherence is contained in the registers. In addition, we lack information on the individuals’ outpatient care. Third, we have no information on the individuals’ socioeconomic status, living conditions and families. Fourth, for legal/ethical reasons, we were unable to access the individuals’ records of arrests, convictions and incarcerations. Fifth, victimization is associated with an increased risk of subsequent violent crime [8, 21]. Incarceration may be an alternative outcome to hospitalization, reducing the number of observed hospitalizations. Sixth, our analyses have not accounted for potential effects of alcohol use disorders. Since there is a 93% treatment gap for alcohol use disorders in the Czech Republic [28], most of the individuals with these disorders are not recorded in health registers. Thus, using register records of alcohol use disorders as a covariate would be inappropriate. These lacking data represent factors that may have affected our results, and thus must be considered unmeasured confounders. Our results need to be replicated, preferably using longitudinal prospective design or utilizing health registers containing information from both in-patient and out-patient care.

## Conclusions

In this study, we showed that individuals with a history of SMI hospitalization were approximately three times more likely to be rehospitalized for SMI within a 6-month post-assault period than their non-assaulted counterparts. A history of comorbid DUD and the number of previous SMI hospitalizations slightly reduced the observed effects. Soon after a person with SMI is physically assaulted, there should be an evaluation by a mental health professional and close follow-up. Medical and psychological support should be provided as needed. After the patient is victimized, care givers should be particularly vigilant regarding the potential for drug and alcohol abuse. This approach could potentially improve the quality of life of individuals with SMI as well as reduce societal and rehospitalization costs, particularly in the 6-month period following an assault.

## Supplementary Information

**Additional file 1. **STROBE checklist

**Additional file 2. **ICD codes for severe mental illnesses, drug use disorders and assaults

**Additional file 3. **Coding procedure

## Data Availability

The data that support the findings of this study can be made available by the corresponding author (KM) upon reasonable request.
