# Supplementary material for: Serious physical assault and subsequent risk for rehospitalization in individuals with severe mental illness: a nationwide, register-based retrospective cohort study
Source: Ann Gen Psychiatry. 2021 Sep 18;20:44. doi: 10.1186/s12991-021-00358-y (PMC8449895; doi:10.1186/s12991-021-00358-y)
Supplement: Supplementary file 2 — Additional file 2. ICD codes for severe mental illnesses, drug use disorders and assaults [file 12991_2021_358_MOESM2_ESM.docx]

| **Severe mental illnesses (SMI) ICD codes** | |
| --- | --- |
| F20.0 – F20.9 | Schizophrenia |
| F25.0 – F25.9 | Schizoaffective disorder |
| F31.0 – F31.9 | Bipolar disorder |
| F32.0 – F32.9 | Major depressive disorder, single episode |
| F33.0 – F33.9 | Major depressive disorder, recurrent episode |

| **Drug use disorders (DUD) ICD codes** | |
| --- | --- |
| F11.0 – F11.9 | Mental and behavioural disorders due to use of opioids |
| F13.0 – F13.9 | Mental and behavioural disorders due to use of sedatives and hypnotics |
| F14.0 – F14.9 | Mental and behavioural disorders due to use of cocaine |
| F15.0 – F15.9 | Mental and behavioural disorders due to use of other stimulants, including caffeine |
| F16.0 – F16.9 | Mental and behavioural disorders due to use of hallucinogens |

| **Assaults ICD codes** | |
| --- | --- |
| X93.0 – X93.9 | Assault by handgun discharge |
| X94.0 – X94.9 | Assault by rifle, shotgun and larger firearm discharge |
| X95.0 – X95.9 | Assault by other and unspecified firearm and gun discharge |
| X99.0 – X99.9 | Assault by sharp object |
| Y00.0 – Y00.9 | Assault by blunt object |
| Y01.0 – Y01.9 | Assault by pushing from high place |
| Y02.0 – Y02.9 | Assault by pushing or placing victim in front of moving object |
| Y03.0 – Y03.9 | Assault by crashing of motor vehicle |
| Y04.0 – Y04.9 | Assault by bodily force |
| Y05.0 – Y05.9 | Sexual assault by bodily force |
