# Supplementary material for: Serious physical assault and subsequent risk for rehospitalization in individuals with severe mental illness: a nationwide, register-based retrospective cohort study
Source: Ann Gen Psychiatry. 2021 Sep 18;20:44. doi: 10.1186/s12991-021-00358-y (PMC8449895; doi:10.1186/s12991-021-00358-y)
Supplement: Supplementary file 3 — Additional file 3. Coding procedure [file 12991_2021_358_MOESM3_ESM.docx]

**The coding procedure**

The ICD codes are assigned by coders on the basis of hospital discharge reports. The coders are MDs or administrative workers with MD backup, and they receive special training for this work. Before starting principal data analyses, we checked the coders’ performance at three university hospitals in different cities (Olomouc, Pilsen, and Prague). Each of these hospitals had one coder. We generated six fictitious vignettes described in the format of discharge reports that were presented to the coders. Five of the vignettes contained various verbal descriptions of assaults by bodily force, plus several other features such as surgical diagnoses of injuries, homelessness and alcohol intoxication. One of the vignettes did not contain a description of assault. The coders’ performance was 100% correct (no false positives or negatives) for assault by bodily force (Y04).
